# Post-Traumatic Stress Disorder after the 6.1 Magnitude Earthquake in Piura, Peru: A Cross-Sectional Study

**DOI:** 10.3390/ijerph191711035

**Published:** 2022-09-03

**Authors:** Mario J. Valladares-Garrido, Luis E. Zapata-Castro, C. Ichiro Peralta, Abigaíl García-Vicente, David Astudillo-Rueda, Darwin A. León-Figueroa, Cristian Díaz-Vélez

**Affiliations:** 1 South American Center for Education and Research in Public Health, Universidad Norbert Wiener, Lima 15046, Peru; 2 Hospital Regional Lambayeque, Chiclayo 14012, Peru; 3 Faculty of Medicine, Universidad Nacional de Piura, Piura 20002, Peru; 4 Sociedad Científica de Estudiantes de Medicina de la Universidad Nacional de Piura (SOCIEMUNP), Piura 20002, Peru; 5 Faculty of Medicine, Universidad Nacional Federico Villarreal, Lima 15088, Peru; 6 Faculty of Medicine, Universidad César Vallejo, Piura 20001, Peru; 7 Emerge, Emerging Diseases and Climate Change Research Unit, School of Public Health and Administration, Universidad Peruana Cayetano Heredia, Lima 15013, Peru; 8 Faculty of Medicine, Universidad de San Martín de Porres, Chiclayo 14012, Peru; 9 School of Medicine, Universidad Privada Antenor Orrego, Trujillo 13008, Peru; 10 Instituto de Evaluación de Tecnologías en Salud e Investigación-IETSI, EsSalud, Lima 15072, Peru

**Keywords:** stress disorders, post-traumatic, earthquakes, prevention and mitigation, disasters

## Abstract

In Peru, major disasters like the 2007 Pisco earthquake have produced high rates of post-traumatic stress. However, evidence is still needed to strengthen interventions. In 2021, a major earthquake struck Piura, in northern Peru. In this context, we aimed to assess the prevalence of PTSD and its associated factors. A cross-sectional study was conducted during August–September 2021 in people who experienced the 6.1 Piura earthquake on 30 July 2021. The questionnaire included the PCL-C, CD-RISC, ISI, HFIAS, and additional demographic data. Generalized linear models were used. The prevalence of PTSD was 20.3%. Household income was between PEN 2001 and 3000 (PR = 4.26, 95% CI: 1.08–16.75), smoking (PR = 2.49, 95% CI: 1.03–6.01), experience of a nervous breakdown (PR = 1.83, 95% CI: 1.09–3.09), moderate food insecurity (PR = 2.91, 95% CI: 1.10–7.73), and severe insomnia (PR = 8.25, 95% CI: 2.22–30.71) increased the prevalence of PTSD. One out of five individuals experienced post-traumatic stress symptoms after the 2021 earthquake in Piura, which varies depending on socioeconomic, psychosocial, and individual factors. Further research should strengthen these findings to ensure a fair and early mental health intervention against new seismic events in this and other Peruvian regions.

## 1. Introduction

Post-traumatic stress disorder (PTSD) is a weakening condition caused by past life-threatening events that manifests as symptoms of re-experiencing, blunting, avoidance, and hyperarousal [1]. This disorder has shown increased rates of severe disability, illness, and premature death [2]. Worldwide, it has been reported that more than two-thirds of the general population experience trauma following an earthquake at some point in their lives, resulting in a wide range of physical and mental health consequences [3]. Previous studies reported that at least 50% of earthquake survivors may develop chronic depression, generalized anxiety, and PTSD [4], the latter being the most prevalent psychiatric morbidity, with symptoms reported between 9 and 57% [5,6]. The difference in these estimates relies on, generally, the magnitude variance of the seismic event, quality of household, social support, and baseline mental health [6,7].

Peru’s location within the ‘‘Pacific Ring of Fire’’, a geographical area characterized by the most important seismic events in the world, is associated with a higher risk of experiencing major earthquakes [8]. Nonetheless, little is evidenced on the mental health impact of survivors. A study reported an increase in the number of psychological consultations, with an incidence of 19% one year after the 7.0 magnitude earthquake occurred in 2005 in San Martin [9]. Additionally, in 2007 after the 7.9 magnitude earthquake in Pisco, the overall prevalence of chronic PTSD was found to be 16%, which was considerably higher than expected [7]. Fourteen years later, a 6.1 magnitude earthquake struck Piura, northern Peru. Material damage was reported and the effect on the mental health of the population [10], but it remains unclear what particular conditions increase PTSD in the long term. This would help to improve disaster preparedness especially in people with little access to mental health services.

Post-earthquake PTSD is generally attributed to high levels of chronic stress due to uncertainty about the immediate future in terms of material, social, and family possessions [11]. This condition is exacerbated by the presence of unfavorable characteristics. For example, a systematic review based on studies from Peru, Taiwan, Turkey, China, Greece, Haiti, Pakistan, Italy, Iran, and Japan has shown that low family income is an important predictor of PTSD [12]. This condition was also more prevalent in survivors suffering from insomnia after the 2008 Wenchuan earthquake [13,14,15] and in those with low resilience after the 2012 Yiliang, 2011 Japan, and 2008 Wenchuan earthquakes [16,17,18]. Inadequate social support was an additional important predictor according to evidence from the 2017 Jiuzhaigou and 2013 Lushan earthquakes [19,20]. Finally, a study after the 2007 earthquake in Pisco, Peru, but also after the 2008 Wenchuan earthquake supported that severe household damage was related to higher rates of post-traumatic stress symptoms [7,21]. To expand this knowledge and promote efficient interventions, we aimed to identify the prevalence and factors associated with PTSD in people affected by the 6.1 magnitude earthquake that occurred in Piura on 30 July 2021.

## 2. Materials and Methods

### 2.1. Study Design

A cross-sectional study was conducted during August–September 2021 in people from Piura who experienced a seismic event of magnitude 6.1 according to the Richter scale on 30 July 2021.

An online survey was designed using REDCap^®^. A Facebook page was created to share survey invitation posts. To this end, ads were promoted using Facebook’s advertising services to reach a larger number of people in Piura. The survey was also disseminated on social networks of local health institutions, universities, and press media (television, radio, and internet), that agreed to share the survey free of charge.

### 2.2. Participants

The population consisted of residents of the department of Piura, Peru, who suffered the earthquake of 30 July 2021, and who lived in 1 of the 38 districts of Piura, declared in a state of emergency after the seismic event.

Individuals over 18 years of age, who experienced the earthquake and resided in Piura, were included. People who did not respond to the variables of interest in the survey were excluded. Also excluded were those who self-reported residing in Piura but were not present during the earthquake.

The estimated sample size was 179, which was based on an expected prevalence of 12%, a confidence level of 95%, a margin of error of 5%, and a refusal rate of 10%. The final sample included a total of 177 participants. Snowball sampling was applied by initially recruiting individuals living in urban and rural areas from the most affected cities of Piura (Piura, Sullana, Chulucanas, Paita, and Colan), and then asking participants to share the survey to contacts in their and other locations. Internet coverage in this Peruvian region is approximately 53% of individuals over 14 years of age. We used this approach to increase the probability of obtaining the minimally required sample size.

### 2.3. Measures

PTSD symptoms were assessed using the PTSD Checklist—Civilian Version (PCL-C). This questionnaire assesses the general traumatic experience, based on the Diagnostic and Statistical Manual of Mental Disorders, Fourth Edition (DSM-IV) criteria, and in accordance with the National Center for PTSD [22]. The PCL-C includes 17 questions rated on a 5-point Likert scale and assesses the domains of trauma re-experiencing (domain B), trauma avoidance and blunting (domain C), and hyperarousal (domain D) [14]. The overall scores range from 17 to 85, with higher scores indicating higher PTSD symptoms [22,23]. The instrument has been used in the context of natural disasters in Peru and validated in the Peruvian and other Spanish-speaking populations, with a Cronbach’s alpha coefficient of 0.94, sensitivity of 95%, and a diagnostic efficacy of 95% [7,24,25,26,27,28]. For the purpose of this study, presence of PTSD symptoms was defined as an overall score > 43 [22,23].

### 2.4. Exposures

Resilience was assessed with the Connor–Davidson Resilience Scale (CD-RISC). The questionnaire has 10 items rated on a 5-point Likert scale [29]. The CD-RISC has been validated in Spanish-speaking health personnel, workers in different occupational fields, and young Spanish adults [30,31,32]. Cronbach’s alpha coefficient was 0.80, sensitivity 70%, and specificity 68.2% in discriminating healthcare workers with depression [29,30,31,32]. The overall scores range from 0 to 40, with higher scores indicating higher resilience levels. For the purpose of this study, high resilience was defined as an overall score > 23 [29,30,31,32].

Insomnia was assessed using the Insomnia Severity Index (ISI). This questionnaire consists of seven items rated on a 5-point Likert scale [33]. The ISI has been validated for the general Spanish-speaking population, with a Cronbach’s alpha coefficient of 0.82 [34]. The scale has been used for the study of insomnia in Latino communities residing in the United States (2156 participants) [35]. The overall scores range from 0 to 28, with higher scores indicating higher insomnia severity. Severity of insomnia is classified as sub-threshold (8–14 points), moderate (15–21 points), and severe (22–28 points) [33]. For the purpose of this study, presence of insomnia was defined as an overall score > 7.

Food security was measured using the Household Food Insecurity Access Scale (HFIAS). The questionnaire consists of 9 items rated on a 3-point Likert scale. The HFIAS has three domains: anxiety and uncertainty about household food supply, food quality/insufficient quality food intake, and physical consequences [36]. The overall score is calculated as the sum of the item scores, with higher scores indicating higher food insecurity. According to the Food and Nutrition Technical Assistance III Project (FANTA III) [36], mild food insecurity occurs with scores ranging from 2 to 3 in item 1, 1 to 3 on item 2, or 1 on items 3 or 4 [36]. Moderate food insecurity occurs with scores ranging from 2 to 3 on items 3 or 4, or 1 to 2 on items 5 or 6. Severe food insecurity occurs with a score of 3 on items 5 or 6, or ranging from 1 to 3 on items 7, 8, and 9 [36]. This scale has been validated for use in Spanish-speaking Latino populations [36].

Sociodemographic and work-related variables were age (years), sex (female, male), marital status (single, married, cohabitant, divorced, separated, widow), educational level (none, initial, primary, secondary, higher non-university, higher university), type of work (worker, maid, student, unemployed, other), monthly household income in Peruvian currency (PEN 300–1000, PEN 1001–2000, PEN 2001–3000, PEN 3001–5000, and PEN 5001 or more), religion (Catholic, non-Catholic, none), and number of family members in the household. Variables related to personal and family medical history were frequent consumption of alcohol and tobacco and comorbidities (none, hypertension, diabetes, obesity, others). Variables related to stressors before, during, and after the earthquake were personal and family history of mental disorders, nervous breakdown immediately after the earthquake, physical injury due to the earthquake, family member with physical injury due to the earthquake, housing damage due to the earthquake (unaffected, mild, moderate, severe), and job loss due to the earthquake. The variable related to social support was social/material support from family, relatives, neighbors, friends, religious members, politicians, government, or non-governmental organizations.

### 2.5. Data Analysis

Sample characteristics were described with frequencies (*n*, %) or the median value and 25–75th percentile.

To compare the frequency of PTSD according to individual characteristics, the chi-squared test was used for categorical variables, while the Mann–Whitney U test for numerical data with a non-normal distribution.

#### Model

To assess the association between PTSD and potential influencing factors, generalized linear models were used with Poisson distribution, robust variance, and log-link function. The unadjusted model equation was as follows:

*Ln* (*λ_j_*) = *β*_0_ + *β*_1_ ∗ *X_j_*

where

*λ_j_*: probability of occurrence of the event given a *j* value.

*β*_0_: intercept, equal to *ln* (*λ_j_*) when *X_j_* = 0.

*β*_1_: coefficient of *X_j_*, equal to *ln* (*λ_j_*_+1_/*λ_j_*) when *j* increases by one unit.

*X_j_*: variable with value *j.*

Variables associated with the outcome in the unadjusted model were included in the adjusted analysis, based on the following equation:

*Ln* (*λ_j_*) = *β*_0_ + *β_i_* ∗ *X_jk_*

where

*λ_j_*: probability of occurrence of the event given a *j* value.

*β*_0_: intercept, equal to *ln* (*λ_j_*) when *X_jk_* = 0.

*β_i_*: coefficient of *X_jk_*, equal to *ln* (*λ_j_*_+1_/*λ_j_*) when *j* increases by one unit and the other *i* − 1 variables remain constant.

*X_jk_*: variable *k* (*k* = 1, 2…, *i*) with value *j*.

Prevalence ratios (PRs) with 95% confidence intervals were reported. *p*-values < 0.05 were considered statistically significant. Collinearity was evaluated between the variables of interest. The analyses were performed with Stata v. 17 (StataCorp, College Station, TX, USA, 2016).

## 3. Results

**Figure 1:**
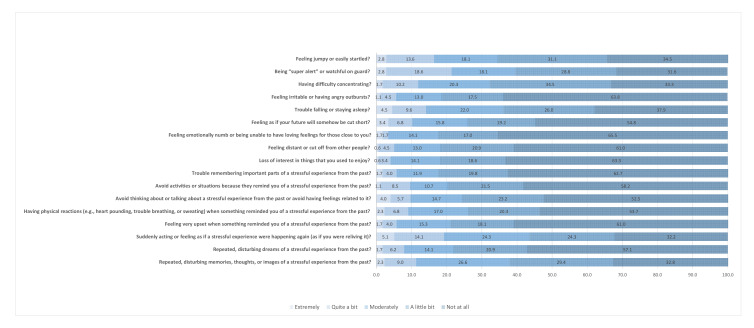
Frequency of PTSD symptoms according to the PCL-C items.

Participant characteristics are presented in Table 1. A total of 177 residents of Piura who were exposed to the 6.1 magnitude earthquake were analyzed. Of the total, 56% (*n* = 98) were female, 64% (*n* = 113) reported having a higher level of education, and 55% (*n* = 98) were currently studying. Seventy six percent (*n* = 134) were at home at the time of the earthquake, and 19% (*n* = 34) reported that their houses had suffered minor damage due to the seismic event. Six percent (*n* = 10) had moderate food insecurity and 7% (*n* = 12) experienced moderate insomnia. Forty one percent (*n* = 71) scored high resilience and 20% (*n* = 36) experienced PTSD symptoms due to the earthquake. The frequency of each PTSD symptom is shown in Figure 1.

Table 2 shows the prevalence of PTSD symptoms according to individual characteristics. High resilience was associated with a lower frequency of PTSD symptoms (11% high resilience vs. 26% low resilience, *p* = 0.021). Severe insomnia was associated with a higher prevalence of PTSD symptoms (75% severe insomnia vs. 10% absent insomnia, *p* < 0.001). Severe food insecurity was associated with a higher frequency of PTSD symptoms (44% severe food insecurity vs. 15% no food insecurity, *p* < 0.001).

To identify potential factors associated with PTSD symptoms, a Poisson regression analysis was performed. All exposures that showed a *p*-value < 0.05 in the simple regression model (unadjusted) was included for the adjusted analysis. A household income between PEN 2001 and 3000 was found to be associated with a higher prevalence of PTSD symptoms (PR = 4.26, 95% CI = 1.08*–*16.75). Smoking (PR = 2.49, 95% CI = 1.03*–*6.01) and severe insomnia (PR = 8.25, 95% CI = 2.22*–*30.71) were associated with a higher frequency of PTSD symptoms. Having suffered a nervous breakdown immediately after the earthquake (PR = 1.83, 95% CI = 1.09*–*3.09), moderate food insecurity (PR = 2.91, 95% CI = 1.10*–*7.73), and social/material support from non-governmental organizations (PR = 4.39, 95% CI = 2.02*–*9.52) were also associated with PTSD symptoms. A high level of resilience (PR = 0.51, 95% CI = 0.27*–*0.95) and having experienced the earthquake in a public place (PR = 0.52, 95% CI = 0.32*–*0.85) were associated with a lower prevalence of PTSD symptoms. More details in Table 3.

## 4. Discussion

### 4.1. Findings

In total, 5 out of 10 participants were found to experience PTSD symptoms. Factors associated with a higher prevalence of PTSD symptoms were an income between PEN 1001 and 2000, smoking, a nervous breakdown immediately after the earthquake, a family member with physical injury due to the earthquake, social/material support from non-governmental organizations, moderate food insecurity, and severe insomnia. Conversely, high resilience and having experienced the earthquake in a public place were associated with a lower frequency of PTSD symptoms.

Approximately 2 out of 10 participants experienced PTSD symptoms due to the 6.1 seismic event. Previous studies in Europe, Asia, and Latin America have documented a prevalence of PTSD ranging from 5 to 60% as a consequence of natural disasters, including earthquakes [37]. In Peru, a frequency of 25% has been reported since 2010, five months after the traumatic event [37], which is a slightly higher value than that found in our study. PTSD is currently considered to be the most prevalent type of psychiatric disorder after disasters caused by catastrophic events or unusual threats [12]. Furthermore, it has been established that the severity of exposure is positively associated with the risk of subsequent PTSD [38] and other mental disorders [12,37]. This finding is similar to the frequency reported before, especially when an assessment was performed 2 months after the stressful event [39]. However, previous studies suggested that the prevalence of PTSD increases months or even several years after the trauma [40]. Similar research reported that 33% of affected individuals may develop more severe PTSD symptoms leading to long-term cognitive and behavioral impairment [40]. It is important to understand the effect of PTSD on mental, social, and physical health in the context of natural disasters to mitigate the impact on quality of life [41].

Lower family income was found to be associated with a higher prevalence of PTSD symptoms. The economic impact of large-scale natural disasters tends to occur long after the immediate aftermath [42]. The notable disparity in the incidence of PTSD symptoms within a population could be explained by the socioeconomic status and resource capacity of individual households [12]. Other influencing factors include the intensity of the earthquake, the amount of property lost, and the economic impact due to the death of family members [12,43]. Previous studies have shown that low-income individuals and those who were economically harmed by a catastrophe are more likely to develop PTSD [42]. The mental health burden can be reduced by support programs, like helping others to restore their housing and resources [42]. Other similar interventions could help people overcome the economic consequences of a seismic event.

Smoking was also found to be associated with a higher frequency of PTSD symptoms. This behavior can contribute to the development of several conditions that impair the ability to work and interact with the environment [44]. The frequency of smoking in people experiencing PTSD is 45%, which is three times the frequency in the general population [45]. There is consistent evidence that smoking contributes to reduced life expectancy among people with PTSD [46]. In particular, intolerance to high levels of distress is considered to be an important factor in sustaining smoking [46]. Therefore, there is a need to better understand the factors that predispose to smoking and the barriers that limit adequate treatment in the context of an earthquake or other natural disaster.

Interestingly, having experienced the earthquake in a public place was found to be associated with a lower prevalence of PTSD. A previous study conducted in Peru four years after the 2007 Pisco earthquake showed no association between PTSD and the location of the earthquake [7]. The association found here could be explained by the intensity of distressing memories generated by constant exposure to the site [47]. The experience of an earthquake at home or at another frequently visited location may increase mental burden depending on the level of trauma experienced [48,49,50,51]. Further evidence is needed to explain these differences.

Having suffered a nervous breakdown immediately after the earthquake was associated with a higher prevalence of PTSD symptoms. Nervous breakdown is associated with temporary inability to react physically and mentally to stressful situations [52]. It may also be associated with panic disorder symptoms [52] that have been frequently reported during traumatic events (41–53%) [53,54]. A previous study found an association between peritraumatic panic and chronic PTSD, which is mediated by peritraumatic dissociation components [55]. This suggests that early detection of peritraumatic reactions would significantly help to identify individuals at risk for PTSD [56].

Participants who had a family member with physical injuries due to the earthquake experienced a higher frequency of PTSD symptoms. This result supports an earlier Peruvian study [7] that found a PTSD prevalence of 11% in people who lost family members during the 2007 Pisco earthquake. In addition, the latter study and another [21] conducted in the 2008 Wenchuan earthquake found the existence of PTSD symptoms in people with physical injuries, although without significant differences with healthy people, which is consistent with what was found in the present study. It is likely that the injury of a family member is more significant for the individual, as it could represent an additional mental burden to that experienced by the earthquake. It is important to identify factors that may mediate the presence of PTSD, such as the degree of social support, economic capacity, or level of resilience.

Unexpectedly, participants who received social/material support from non-governmental organizations experienced a higher prevalence of PTSD. This is different from one study [19] showing that quality of social support was associated with reduced levels of PTSD and accounted for an additional 2% of the variance over other predictors, including quantity of social support [19,20]. This result should be interpreted with caution due to the small sample size. However, at least in our setting, social support from non-governmental organizations seems to be unequal and does not reach all people affected by a natural disaster. Therefore, this result should be further validated to ensure the provision of material and other support for a fair response in emergency situations.

Severe insomnia was found to be associated with a higher prevalence of PTSD symptoms. This is consistent with a cohort study reporting that adolescent earthquake survivors diagnosed with PTSD were 50% more likely to have insomnia [13]. Another study found that the association of PTSD and insomnia is mediated by resilience [14]. In contrast, other studies [13,15] indicated that insomnia is a predictor of PTSD. The complex relationship between insomnia and PTSD may be related to the influence of sleep on memory consolidation and emotion regulation [57]. Consequently, further studies should provide better evidence in contexts such as earthquakes and other natural disasters.

Finally, high resilience was found to be associated with a lower frequency of PTSD. This is similar to a study after the 2011 earthquake in Japan that found a 50% lower prevalence of PTSD in people with high resilience [16]. Similarly, a study following the 2008 Wenchuan earthquake showed a negative association between a resilient personality and the development of PTSD [17]. However, there are other studies that found no statistically significant differences [18]. Although these reports used the same scale as the present study, it is likely that populations have different cultural traits that determine a different pattern of response to a catastrophe. More evidence on earthquake-affected populations is needed to support this hypothesis.

### 4.2. Recommendations

The present study is the first to explore factors influencing the development of post-traumatic stress after the Piura earthquake in 2021. Although our results add to previous Peruvian and Latin American evidence on post-disaster mental disorders [7,37,58,59], accurate information for public health policies is still needed. Mental health interventions in Peru are provided by the Ministry of Health in emergency and disaster situations [60]. Nonetheless, these interventions should be improved in light of valid and up-to-date scientific evidence to allow for timely assessment and treatment of PTSD. For example, we evidenced that special attention should be given to vulnerable populations in Piura [61], such as those exposed to food insecurity and low household income. Additionally, individuals with prior psychiatric disorders, such as insomnia, are a target for interventions as they may be at higher risk for post-disaster PTSD. Early management of post-traumatic stress in this context can help to predict and prevent other serious outcomes, such as impaired quality of life, depression, and suicide.

### 4.3. Limitations

The study has important limitations. First, the sample size was smaller than estimated and regression analysis was therefore underpowered. Second, the cross-sectional design of the study does not allow us to establish a causal relationship between the study variables. Third, the non-probabilistic sampling limits the inference of results to the entire population of Piura. Fourth, the data collection strategy may have biased the selection of participants, as young people frequently use social media and the internet. Fifth, common survey-related biases, such as recall or social acceptability bias, may have affected the accuracy of all responses. Sixth, some included variables (i.e., insomnia, nervous breakdown, family member injured, housing damage, job loss, and material/social support) may have acted as either exposure to or outcome of PTSD due to assessment at one point in time. Seventh, we used a version of PCL-C based on the DSM-IV criteria, so the prevalence of PTSD may have been different compared to the updated DSM-V version. Despite these limitations, the questionnaire was administered within the first two months after the earthquake; therefore, the mental health variables were measured in a period near to the traumatic exposure. Additionally, the instruments were previously validated and showed high internal consistency.

## 5. Conclusions

We conducted an online survey study in the general population of Piura after the 6.1 magnitude earthquake in 2021. Our findings indicate that one out of five individuals have experienced post-traumatic stress symptoms, and those with more vulnerable characteristics, such as low family income, food insecurity, smoking, and insomnia, were more likely to experience PTSD. The prevalence of post-disaster PTSD found in this study is in line with the current global evidence. Notably, it was very similar to that found in an earlier study following one of Peru’s most devastating earthquakes during the 2007 in Pisco [7]. In addition, the main factors associated with PTSD identified here are consistent with the majority of previous similar studies. However, some significant variables, such as support from non-governmental organizations and the location where the earthquake was experienced, should be further explored as no comparable study has been found to date.

It is important to note that data collection was carried out mainly in the most affected cities through snowball sampling and using validated instruments (e.g., PCL-C, CD-RISC, ISI, HFIAS). In addition, generalized linear modeling helped to identify variables independently associated with PTSD. Future research should confirm our findings by overcoming current limitations, but also by focusing on testing the effectiveness of interventions to reduce PTSD in higher-risk groups. The timely management of this condition would ensure fair mental health care in the face of new seismic events in this and other regions of Peru.

## Figures and Tables

**Table 1:** Participant characteristics (*n* = 177).

Characteristics		*n* (%)
Age *		22 (20–29)
Sex		
	Female	98 (56.0)
	Male	77 (44.0)
Marital status		
	Single	140 (79.1)
	Married	25 (14.1)
	Cohabitant	10 (5.7)
	Divorced	1 (0.6)
	Separated	1 (0.6)
Education level		
	Secondary	46 (26.0)
	Higher non-university	18 (10.2)
	Higher university	113 (63.8)
Current work		
	Worker	50 (28.3)
	Maid	9 (5.1)
	Student	98 (55.4)
	Unemployed	7 (4.0)
	Others	13 (7.3)
Household income (USD)		
	75–250	28 (15.8)
	251–500	65 (36.7)
	501–750	22 (12.4)
	751–1250	31 (17.5)
	1251 or more	31 (17.5)
Religion		
	Catholic	143 (80.8)
	Non-Catholic	17 (9.6)
	None	17 (9.6)
Family members (number) *		4 (3–5)
Alcoholism (Yes)		17 (9.8)
Smoking (Yes)		11 (6.3)
Comorbidity		
	No	147 (84.5)
	Hypertension	4 (2.3)
	Diabetes	2 (1.2)
	Obesity	14 (8.1)
	Other	7 (4.0)
Personal history of mental disorders (Yes)		12 (6.8)
Family history of mental disorders (Yes)		20 (11.3)
Place where the person experienced the earthquake		
	Home	134 (75.7)
	Neighbor’s/friend’s house	5 (2.8)
	Workplace	15 (8.5)
	Public space	23 (13.0)
Nervous breakdown immediately after the earthquake (Yes)		69 (39.0)
Physical injury due to the earthquake (Yes)		5 (2.8)
Family member with physical injury due to the earthquake (Yes)		3 (1.7)
House damage due to the earthquake		
	Not affected	140 (79.1)
	Mild	34 (19.2)
	Moderate	2 (1.1)
	Severe	1 (0.6)
Job loss due to the earthquake (Yes)		4 (2.3)
Social or material support		
	Family/relatives	105 (59.3)
	Neighbors	38 (21.6)
	Friends	93 (52.8)
	Religious members	17 (9.7)
	Politicians	11 (6.3)
	Government	10 (5.7)
	Non-governmental organizations	6 (3.4)
Food insecurity		
	No	122 (68.9)
	Mild	29 (16.4)
	Moderate	10 (5.7)
	Severe	16 (9.0)
Insomnia		
	Absent	94 (55.6)
	Sub-threshold	59 (34.9)
	Moderate	12 (7.1)
	Severe	4 (2.4)
Resilience		
	Low	102 (59.0)
	High	71 (41.0)
Post-traumatic stress disorder		
	No	141 (79.7)
	Yes	36 (20.3)

* Median (percentile 25–percentile 75).

**Table 2:** Characteristics associated with PTSD.

Variables		Post-Traumatic Stress Disorder		p *
		No (*n* = 141)	Yes (*n* = 36)	
		*n* (%)	*n* (%)	
Age †		22 (20–27)	22 (21–32)	0.500 ‡
Sex				0.196
	Female	75 (76.5)	23 (23.5)	
	Male	65 (84.4)	12 (15.6)	
Single marital status		111 (79.3)	29 (20.7)	0.809
Education level				0.52
	Secondary	35 (76.1)	11 (23.9)	
	Higher non-university	16 (88.9)	2 (11.1)	
	Higher university	90 (76.7)	23 (20.4)	
Current work				**0.018**
	Worker	38 (76.0)	12 (24.0)	
	Maid	4 (44.4)	5 (55.6)	
	Student	84 (85.7)	14 (14.3)	
	Unemployed	4 (57.1)	3 (42.9)	
	Others	11 (84.6)	2 (15.4)	
Household income (USD)				0.34
	75–250	25 (89.3)	3 (10.7)	
	251–500	47 (72.3)	18 (27.7)	
	501–750	17 (77.3)	5 (22.7)	
	751–1250	26 (83.9)	5 (16.1)	
	1251 or more	26 (83.9)	5 (16.1)	
Religion				0.633
	Catholic	113 (79.0)	30 (21.0)	
	Non-Catholic	13 (76.5)	4 (23.5)	
	None	15 (88.2)	2 (11.8)	
Household members (number) †		4 (4–6)	4 (3–5)	0.381 ‡
Alcoholism (Yes)		12 (70.6)	5 (29.4)	0.314
Smoking (Yes)		6 (54.6)	5 (45.5)	**0.03**
Comorbidity (Yes)		22 (81.5)	5 (18.5)	0.822
Personal history of mental disorders (Yes)		8 (66.7)	4 (33.3)	0.247
Family history of mental disorders (Yes)		13 (65.0)	7 (35.0)	0.084
Place where the person experienced the earthquake				0.374
	Home	109 (81.3)	25 (18.7)	
	Neighbor’s/friend’s house	3 (60.0)	2 (40.0)	
	Workplace	10 (66.7)	5 (33.3)	
	Public place	19 (82.6)	4 (17.4)	
Nervous breakdown immediately after the earthquake (Yes)		46 (66.7)	23 (33.3)	**0.001**
Physical injury due to the earthquake (Yes)		46 (66.7)	23 (33.3)	**0.001**
Family member with physical injury due to the earthquake (Yes)		1 (33.3)	2 (66.7)	**0.044**
House damage due to the earthquake				**0.025**
	Not affected	117 (83.6)	23 (16.4)	
	Mild	23 (67.7)	11 (32.4)	
	Moderate	1 (50.0)	1 (50.0)	
	Severe	0 (0.0)	1 (100.0)	
Job loss due to the earthquake (Yes)		2 (50.0)	2 (50.0)	0.136
Social or material support				
	Family/relatives	85 (81.0)	20 (19.1)	0.606
	Neighbors	30 (79.0)	8 (21.1)	0.839
	Friends	76 (81.7)	17 (18.3)	0.572
	Religion members	14 (82.4)	3 (17.7)	0.808
	Politicians	8 (72.7)	3 (27.3)	0.526
	Government	7 (70.0)	3 (30.0)	0.409
	Non-governmental organizations	4 (66.7)	2 (33.3)	0.401
Food insecurity				**0.004**
	No	104 (85.3)	18 (14.8)	
	Mild	23 (79.3)	6 (20.7)	
	Moderate	5 (50.0)	5 (50.0)	
	Severe	9 (56.3)	7 (43.8)	
Insomnia				**<0.001**
	Absent	85 (90.4)	9 (9.6)	
	Sub-threshold	42 (71.2)	17 (28.8)	
	Moderate	8 (66.7)	4 (33.3)	
	Severe	1 (25.0)	3 (75.0)	
Resilience				**0.021**
	Low	76 (74.5)	26 (25.5)	
	High	63 (88.7)	8 (11.3)	

* *p*-value calculated with the chi-squared test; † Median (percentile 25–percentile 75); ‡ *p*-value calculated with the Mann–Whitney U test. Bold numbers: to emphasize statistically significant variables (*p* < 0.05) for the lector.

**Table 3:** Factors independently associated with PTSD.

Variables		Unadjusted			Adjusted		
		PR	95% CI	*p* *	PR	95% CI	*p* *
Age		1.01	0.98–1.03	0.733			
Sex							
	Female	Ref.			Ref.		
	Male	0.66	0.45–0.97	**0.034**	0.99	0.53–1.87	0.992
Single		1.09	0.55–2.18	0.797			
Education level							
	Secondary	Ref.					
	Higher non-university	0.46	0.18–1.17	0.105			
	Higher university	0.85	0.44–1.63	0.627			
Current work							
	Worker	Ref.			Ref.		
	Maid	2.31	1.47–3.64	**<0.001**	1.41	0.32–6.29	0.652
	Student	0.60	0.33–1.08	0.090	0.56	0.31–1.02	0.058
	Unemployed	1.79	0.69–4.64	0.234	2.51	0.27–23.27	0.417
	Others	0.64	0.19–2.16	0.473	1.06	0.47–2.39	0.885
Household income (USD)							
	75–250	Ref.			Ref.		
	251–500	2.59	1.16–5.74	**0.020**	3.33	1.03–10.70	**0.044**
	501–750	2.12	0.87–5.17	0.098	4.26	1.08–16.75	**0.038**
	751–1250	1.50	0.49–4.66	0.478	2.83	0.57–14.07	0.204
	1251 or more	1.50	0.59–3.86	0.394	2.46	0.79–7.67	0.121
Religion							
	Catholic	Ref.					
	Non-Catholic	1.12	0.75–1.68	0.580			
	None	0.56	0.10–3.02	0.500			
Household members		0.92	0.81–1.04	0.194			
Alcoholism		1.54	0.70–3.39	0.284			
Smoking		2.47	1.70–3.59	**<0.001**	2.49	1.03–6.01	**0.042**
Comorbidity		0.91	0.58–1.42	0.669			
Personal history of mental disorders		1.72	0.72–4.07	0.219			
Family history of mental disorders		1.89	0.91–3.93	0.086			
Place where the person experienced the earthquake							
	Home	Ref.			Ref.		
	Neighbor’s/friend’s house	2.14	0.76–6.05	0.149	0.96	0.07–13.51	0.975
	Workplace	1.79	1.05–3.04	**0.032**	1.08	0.45–2.62	0.858
	Public place	0.93	0.38–2.27	0.877	0.52	0.32–0.85	**0.009**
Nervous breakdown immediately after the earthquake		2.77	1.26–6.10	**0.011**	1.83	1.09–3.09	**0.023**
Physical injury due to the earthquake		2.02	0.72–5.68	0.181			
Family member with physical injury due to the earthquake		3.41	1.39–8.35	**0.007**	4.37	1.14–16.73	**0.032**
House damage due to the earthquake							
	Not affected	Ref.			Ref.		
	Mild	1.97	0.99–3.91	0.053	1.46	0.37–5.68	0.587
	Moderate	3.04	0.70–13.18	0.137	4.44	0.82–23.95	0.083
	Severe	6.09	3.82–9.70	**<0.001**			
Job lost due to the earthquake		2.54	0.76–8.47	0.128			
Social or material support							
	Family/relatives	0.86	0.54–1.35	0.509			
	Neighbors	1.08	0.65–1.77	0.772			
	Friends	0.84	0.59–1.20	0.338			
	Religious members	0.88	0.36–2.11	0.769			
	Politicians	1.41	0.53–3.74	0.494			
	Government	1.56	0.66–3.69	0.316			
	Non-governmental organizations	1.72	1.24–2.37	**0.001**	4.39	2.02–9.52	**<0.001**
Food insecurity							
	No	Ref.			Ref.		
	Mild	1.40	0.63–3.12	0.407	1.19	0.67–2.14	0.552
	Moderate	3.39	1.61–7.11	**0.001**	2.91	1.10–7.73	0.032
	Severe	2.97	1.94–4.54	**<0.001**	1.56	0.51–4.78	0.432
Insomnia							
	Absent	Ref.			Ref.		
	Sub-threshold	3.01	1.27–7.16	**0.013**	1.64	0.60–4.50	0.335
	Moderate	3.48	1.19–10.18	**0.023**	2.51	0.51–12.42	0.258
	Severe	7.83	3.88–15.81	**<0.001**	8.25	2.22–30.71	**0.002**
Resilience							
	Low	Ref.			Ref.		
	High	0.44	0.25–0.79	**0.006**	0.51	0.27–0.95	**0.033**

* *p*-values obtained with generalized linear models, Poisson distribution, log-link function, robust variance, and clustering by districts. Bold numbers: to emphasize statistically significant variables (*p* < 0.05) for the lector.

## Data Availability

Not applicable.
